# The Relationship Between Erythropoietin Resistance and Antibody Response to Hepatitis B Vaccine in Hemodialysis Patients

**DOI:** 10.5812/numonthly.8919

**Published:** 2013-05-28

**Authors:** Baris Afsar

**Affiliations:** 1 Department of Medicine, Division of Nephrology, Konya Numune State Hospital, Konya, Turkey

**Keywords:** Erythropoietin, Hepatitis B, Renal Dialysis, Vaccines

## Abstract

**Background:**

Seroconversion following Hepatitis B virus (HBV) vaccine in hemodialysis (HD) patients has been shown to be suboptimal. Nutritional and immunological factors were shown to influence the seroconversion related to HBV vaccination in HD patients. Resistance to erythropoiesis stimulating agents (ESA) for correction of anemia has also been shown to be associated with nutrition and inflammation in these patients.

**Objectives:**

The aim of the current study was to analyze the relationship between anti-HBs response and erythropoietin (EPO) resistance in HD patients.

**Patients and Methods:**

Demographics, clinical characteristics, laboratory parameters and the data about vaccination status were obtained from dialysis charts and vaccination registries retrospectively. To calculate the EPO resistance ESA hypo responsiveness index (EHRI) was used. The EHRI was calculated through deviding the weekly dose of EPO by per kilogram of body weight divided by the hemoglobin level. Patients were divided into non-seroconversion (anti-HBs titers were < 10 IU/L) and seroconversion groups (anti-HBs titers were ≥ 10 IU/L) after completion of the four-dose vaccination schedule.

**Results:**

In total 97 patients were enrolled. For the entire group, stepwise linear regression analysis revealed that square root transformed anti-HBs levels were independently associated with age (P = 0.016), blood urea nitrogen (P = 0.019), high sensitive C-Reactive Protein (P = 0.009), and square root transformed EHRI (P = 0.019). Logistic regression analysis have also demonstrated that blood urea nitrogen (P = 0.002), creatinine (P = 0.046), albumin (P = 0.01) and square root transformed EHRI (P = 0.011) were independently related to seroconversion.

**Conclusions:**

EPO resistance was negatively associated with anti-HBs levels and seroconversion. More studies are needed to highlight the underlying mechanisms regarding EPO resistance and response to HBV vaccination in HD patients.

## 1. Background

Patients on hemodialysis (HD) are at a relatively high risk for exposure to hepatitis B virus (HBV) infection. Therefore, vaccination against HBV has been strongly recommended for the prevention of infection in HD patients. However, despite the availability of vaccination programs, antibody production against HBV surface antigen (anti-HBs) in patients with chronic renal disease is suboptimal. While the percentage of seroconversion following HBV vaccination is 90% in healthy individuals, it is only 50–70% in HD patients (1). Various factors such as genetic predisposition, age, gender, obesity, smoking and concurrent illness has been recognized as possible causes of low responsiveness to HBV vaccine (2-4). Apart from these factors, indices of nutritional status (1, 5) and immune status (6-10) have been shown to influence the seroconversion related to HBV vaccination.

Anemia is a common complication in HD patients and is characterized by a relative deficiency of erythropoietin (EPO) secretion from the diseased kidney relative to the degree of anemia. Therefore, EPO therapy has become the standard treatment in HD patients. Although most HD patients respond adequately to erythropoiesis stimulating agents (ESA), some of them did not respond well to ESA; so-called ESA resistance (11). ESA resistance is defined as a failure to achieve target hemoglobin/ hematocrit levels despite a higher than usual dose of ESA, or a continuous need for this higher dose to maintain target hemoglobin/hematocrit levels (12). In recent studies, the ESA hyporesponsiveness index (EHRI), calculated as the weekly dose of EPO divided by per kilogram of body weight divided by the hemoglobin level (g/dL) has been considered useful to assess the EPO resistance. The EHRI can be easily calculated in the clinic and was directly related to co morbidity and mortality in patients on hemodialysis (HD) (13, 14). It has been clearly shown that one of the most important factors for erythropoietin resistance is the presence of malnutrition and inflammation in HD patients (11, 15, 16).

## 2. Objectives

Since both responses to HBV vaccination and EPO resistance were related with nutritional and inflammatory status in HD patients, it could be possible that these conditions could be interrelated. Thus the current study has been performed to analyze the relationship between anti-HBs response and EPO resistance in HD patients.

## 3. Patients and Methods

This is a retrospective study of in center HD patients who were followed at least for 12 months in the dialysis unit of a state hospital. Baseline demographic data including age, sex, etiologies of kidney disease, type of HD access, presence of diabetes, presence of coronary artery disease were collected. In addition, laboratory results (except from ferritin and parathyroid hormone), kinetic urea modeling for calculation of dialysis dose and total erythropoietin dosage were also examined over the duration of vaccination administration (at 0, 1, 2 and 6 months except from ferritin and parathyroid hormone which were analyzed 0, 3 and 6 months) and the mean of these parameters were used for the final analysis. Body mass index (BMI) was calculated as the ratio of dry weight in kilograms (end-dialysis weight) to height squared (in square meters). Each session lasted for 4–5 h for all patients with blood flow rates of 300–400 mL/min using standard bicarbonate dialysis solution. All patients were virtually anuric and clinically euvolemic. Urea kinetic modeling was performed in order to assess the delivered dose of dialysis using the formula:

spKt/V: −Ln (R − 0.008 × t) + (4 − [3.5 × R]) XUF/W

Where spKt/V is a single-pool Kt/V, R is the ratio of post-dialysis to pre-dialysis serum urea nitrogen, t is the time on dialysis in hours, UF is the amount of ultrafiltration in liters and W is the post-dialysis body weight in kilograms.

Total EPO dose was also recorded for the patients. All the patients were using erythropoietin alpha or erythropoietin beta as ESA. None of the patients received darbepoetin during the study period. All of the patients received recombinant hepatitis B vaccine, given intramuscularly in the deltoid muscle with double doses (40mcg) in a fourth-dose schedule at 0, 1, 2 and 6 months. The data about vaccination status were obtained from dialysis charts and vaccination registries. Immunogenicity or antibody response was determined by the levels of anti-HBs within 1-3 months after the last dose of vaccine. After the initial check for anti-HBs levels, regular anti-HBs levels were checked every 6 months. Booster doses were applied to patients whose anti-HBs levels were decreased below the protective level. Anti-HBs titers were measured using a commercially available enzyme immunoassay (Bioelisa, AntiHb-sAg, Biokit, Barcelona, Spain). We separated the patients into non-seroconversion and seroconversion groups according to their anti-HBs titers: the non- seroconversion group consists of patients whose anti-HBs titers were < 10 IU/L after completion of the four-dose vaccination schedule (this group also involves patients who were nonresponsive to booster doses) and the seroconversion group consists of patients whose anti-HBs titers were ≥ 10 IU/L (this group also involves patients whose anti-HBs levels decreased below the protective levels by time, but responded to a booster dose).

### 3.1. Statistics

Statistical analysis was performed with the SPSS software (Statistical Package for the Social Sciences, version 15.0, SSPS Inc.,Chicago, Ill, USA). Data were shown as mean, standard deviation or percentage where appropriate. Results were considered statistically significant if the 2-tailed P value was < 0.05. Data was checked for normality. Comparisons of the groups were assessed by means of the Student’s T-test for normally distributed variables and by the Mann-Whitney U test for non-normally distributed variables. For the analysis of EHRI levels between the 3 groups; Kruskal-Wallis test was used. For the post hoc analysis of EHRI between these groups; Bonferroni corrected Mann-Whitney U test was used. For the analysis of categorical variables, we used the Chi-Square test and Fisher’s exact test as appropriate. Pearson correlation coefficient r and Spearman correlation coefficient rho were used for the correlation of normally and non-normally distributed variables respectively. Stepwise linear regression was used for the analysis of independent factors (age, gender, HD duration, BMI, presence of smoking, presence of diabetes mellitus, spKt/V, blood urea nitrogen, creatinine, albumin, High sensitive C reactive protein (Hs-CRP) and square root transformed EHRI, related with square root transformed anti-HBs levels (as a dependent parameter)). Logistic regression analysis was also used with the same independent parameters to determine anti-HBs response status (seroconversion vs. non-seroconversion groups, as the dependent variable).

## 4. Results

**Figure 1.**
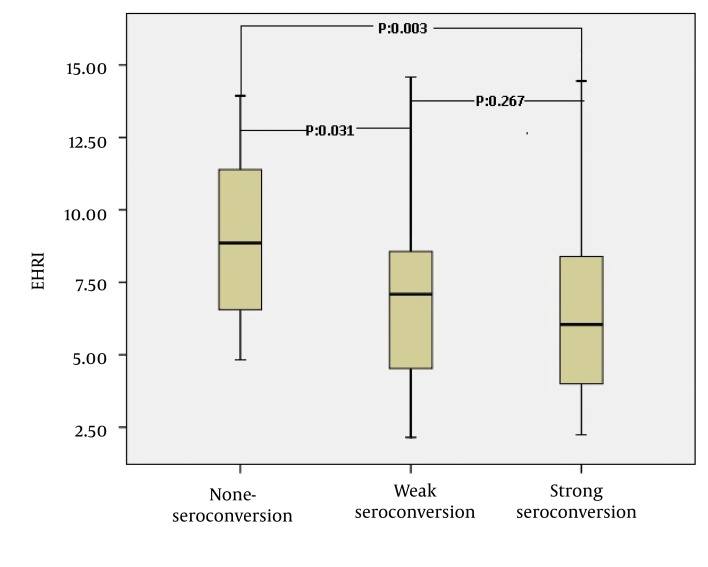
The Comparision of Erythropoiesis Stimulating Agents Hyporesponsiveness Index Among Hemodialysis Patients With Non-Seroconversion, Weak Seroconversion and Strong Seroconversion

Initially 140 patients were enrolled. The inclusion criterion was stable HD patients who were on HD treatment for at least of 12 months. The exclusion criteria were lack of regular 4 doses of vaccination (15 patients), incomplete laboratory data (14 patients), transfer to other centers (5 patients), anti-HCV positivity (4 patients), iron deficiency (5 patients) (defined as a serum ferritin level of < 200 ng/mL or transferring saturation < 20%). There were no patients in our study with a malignancy, showing antibodies against human immunodeficiency virus or taking intradialytic nutritional support during the study period. The final patient population composed of 97 patients. Etiologies for ESRD were as follows; diabetes mellitus in 18, hypertension in 21, glomerulonephritis in 14, vesicourethral reflux and pyelonephritis in 12, nephrolithiasis in 7, polycystic kidney disease in 4, amyloidosis in 4, systemic lupus erythematosus in 1 and unknown in 16 patients. The HD access was the arterio-venous fistula for 79 patients, arterio-venous graft for 11 patients and central venous catheters for 7 patients. Among the 97 patients, 21 (21.6%) comprised the non-seroconversion group, whereas 76 patients (78.4%) comprised the seroconversion group. The comparative sociodemographic and laboratory characteristics of patients with seroconversion and non- seroconversion are given in Table 1. The patients were further divided into 3 groups according anti-HBs levels: Group 1 (n = 21): anti-HBs titer ≤ 10 mIU/mL (non-seroconversion group). Group 2 (n = 29): anti-HBs titer between 10–99 mIU/mL (weak seroconversion). Group 3 (n = 47): anti-HBs titer > 100 mIU/mL (strong seroconversion). The mean of EHRI were 9.19 ± 2.85, 7.30 ± 3.22 and 6.68 ± 3.59 in the 3 groups respectively (P = 0.008). Post hoc analysis of the three groups revealed that Groups 1 and 2 were different with respect to EHRI (P = 0.031) as with group 1 and 3 (P = 0.003). However the EHRI were not different between group 2 and 3 (P = 0.297) (Figure 1).

**Table 1.** The Comparative Sociodemographic and Laboratory Characteristics of Patients With and Without Seroconversion

Parameters	Seroconversion Group (n = 76)	Non-seroconversion Group (n = 21)	P value
**Age, y ^a^**	46.8 ± 14.3	52.2 ± 6.1	0.012 ^b^
**Male/Female, No. ^c^**	48/28	11/10	0.371 ^d^
**Hemodialysis duration, mo ^a^**	86.9 ± 53.7	81.9 ± 52.4	0.706 ^e^
**Body Mass Index, kg/m^2 a^**	23.1 ± 3.5	24.4 ± 5.0	0.249 ^e^
**Smoker/non smoker, No.**	27/49	8/13	0.828 ^d^
**Previous renal transplantation (present/absent), No.**	16/60	1/20	0.109 ^f^
**Diabetes mellitus (present/absent), No.**	13/63	5/16	0.530 ^f^
**Coronary artery disease (present/absent), No.**	21/55	9/12	0.181 ^d^
**Hemoglobin, g/L ^a^**	104.1 ± 9.7	103.6 ± 7.9	0.797 ^c^
**Blood urea nitrogen, mmol/L^a^**	25.8 ± 5.1	21.5 ± 5.9	0.009 ^e^
**Creatinine, µmol/L^a^**	804.4 ± 212.2	760.2 ± 150.3	0.360 ^e^
**Albumin, g/L ^a^**	38.7 ± 5.7	34.3 ± 6.8	0.004 ^c^
**Calcium, mmol/L ^a^**	2.21 ± 0.16	2.15 ± 0.22	0.219 ^b^
**Phosphorus, mmol/L ^a^**	1.67 ± 0.41	1.52 ± 0.23	0.213 ^e^
**Alanine aminotransferase, µkat/L ^a^**	0.27 ± 0.08	0.26 ± 0.13	0.478 ^e^
**Aspartate aminotransferase, µkat/L ^a^**	0.27 ± 0.18	0.27 ± 0.09	0.316 ^e^
**Total Cholesterol, mmol/L ^a^**	4.48 ± 1.03	4.42 ± 0.88	0.979 ^e^
**HDL ^c^-Cholesterol, mmol/L ^a^**	1.08 ± 0.33	1.17 ± 0.27	0.265 ^e^
**LDL ^c^-Cholesterol, mmol/L ^a^**	2.58 ± 1.05	2.40 ± 0.71	0.568 ^e^
**Triglyceride, mmol/L ^a^**	1.88 ± 0.92	2.10 ± 0.91	0.457 ^e^
**Intact Parathyroid Hormone, pg/mL ^a^**	200.5 ± 179.4	201.9 ± 160.4	0.716 ^e^
**Serum Iron, µmol/L ^a^**	10.76 ± 5.34	11.35 ± 5.69	0.668 ^e^
**Ferritin, ng/mL ^a^**	336.8 ± 168.8	307.2 ± 197.0	0.220 ^e^
**Hs-CRP^b^, mg/dL ^a^**	6.62 ± 8.30	3.73 ± 4.84	0.262 ^e^
**spKt/V ^a^**	1.37 ± 0.19	1.32 ± 0.19	0.272 ^b^
**EHRI ^c^**	6.91 ± 3.44	9.19 ± 2.85	0.003 ^e^

^a^mean ± Standart Deviation

^b^P value is based on Student t test

^c^Abbreviations: EHRI, Erythropoiesis stimulating agent hyporesponsiveness index; HDL, High density lipoprotein; Hs-CRP, High Sensitive C-reactive protein

^d^P value is based on chi square test

^e^P value is based on Mann whitney test

^f^P value is based on Fishers exact test

**Figure 2.**
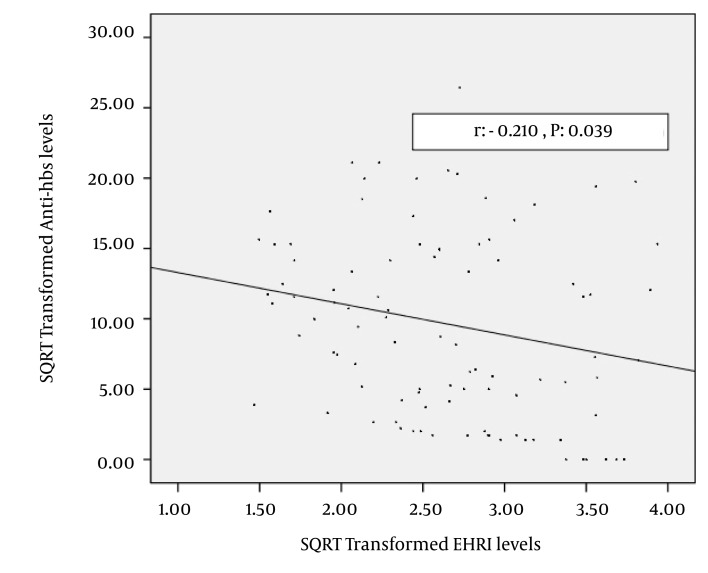
The Scatter Plot Graphic Between Square Root Transformed Erythropoiesis Stimulating Agents Hyporesponsiveness Index and Square Root Transformed Anti-Hbs Levels

For the entire group, spearman correlation analysis revealed that anti-HBs titers were correlated with age: (rho: -0.265, P = 0.009), blood urea nitrogen (rho: 0.290, P = 0.004) albumin, (rho: 0.206, P = 0.043), EHRI (rho: -0.249, P = 0.014) and total EPO dose (rho: -0.206, P = 0.043). The correlation of square root transformed anti-HBs and square root transformed EHRI are shown in Figure 2 (r: -0.210, P = 0.039). Stepwise linear regression of independent factors (as mentioned above) related with square root transformed anti-HBs levels are shown in Table 2. The logistic regression analysis of independet factors related with seroconversion vs. non-seroconversion was given in Table 3.

**Table 2.** Stepwise Linear Regression of Independent Factors Related With Squarerroot Transformed Anti-Hbs Levels

	B^a^	Beta^b^	Confidence Interval	P value
**Constant **	13.337	-	3.567-23.106	0.008
**Age**	-0.119	-0.233	-0.216-(-0.023)	0.016
**Blood Urea Nitrogen**	0.099	0.227	0.017-0.181	0.019
**High Sensitive C-Reactive Protein**	0.214	0.246	0.055-0.373	0.009
**Square root Transformed ** **EHRI** **^c^**	-2.318	-0.219	-4.250-(-0.386)	0.019

^a^B, regression coefficient

^b^Beta, Partial correlation coefficient

^c^EHRI, erythropoiesis stimulating agents hyporesponsiveness index

**Table 3.** Logistic Regression Analysis of Independent Factors Related With Seroconversion

	Exp (B)^a^	95.0 % Confidence Interval for EXP (B)	P value
**Age **	0.955	0.898-1.016	0.143
**Gender (being female)**	0.178	0.029-1.116	0.065
**HD duration**	1.004	0.990-1.019	0.561
**Body Mass Index**	0.848	0.686-1.050	0.130
**Presence of Diabetes**	0.490	0.090-2.673	0.411
**Smoking **	0.191	0.036-1.017	0.052
**Blood Urea Nitrogen**	1.133	1.047-1.226	0.002
**Creatinine**	1.811	1.011 3.246	0.046
**Albumin**	7.002	1.580-31.025	0.01
**High Sensitive C-Reactive Protein**	1.051	0.948-1.164	0.346
**Square root Transformed ** **EHRI** **^b^**	0.184	0.050-0.684	0.011
**spKt/V**	5.237	0.090-9.987	0.425

^a^Exp (B), Odds ratio

^b^Abbreviation: EHRI, erythropoiesis stimulating agents hyporesponsiveness index

## 5. Discussion

In the current study, it was firstly demonstrated that EPO resistance, as evaluated by EHRI, was negatively associated with both anti-Hbs levels (taking as a continuous variable) and seroconversion status (taking as a categorical variable) in stable HD patients.

It is well known that in HD patients anti-HBs levels achieved after HBV vaccination is suboptimal (1). Various factors such as genetic predisposition, age, gender, obesity, smoking and concurrent illness have been recognized as possible causes for none or low responsiveness to HBV vaccine (2-4). Apart from these factors, nutritional status (1, 5) and immune status (6-10) have also influenced response rates. Anemia which is a common condition in HD patients is mostly due to relative deficiency of EPO secretion from the diseased kidney relative to the degree of anemia. Therefore, EPO therapy has become the standard treatment for the anemia of CKD (chronic kidney disease). Although most HD patients respond adequately to ESA, some of them did not respond well to ESA; so-called ESA resistance (11). It was clearly shown that one of the most important factors for erythropoietin resistance is the presence of malnutrition and inflammation in HD patients (11, 15, 16). Since response to HBV vaccination and EPO resistance were related with nutritional and inflammatory status in HD patients, it could be possible that these conditions (vaccination response to HBV and EPO resistance) could be interrelated. Indeed the current study has shown that as EPO resistance (evaluated by EHRI) increased; response to HBV vaccination decreased. Previously, only one study has shown that EPO therapy did not significantly influence antibody responses to immunization with HBV vaccine. However, the authors took EPO therapy as a categorical variable and did not calculate EPO resistance. Additionally, they did not specifically address the anti-Hbs levels (10).

Why EPO resistance and response to HBV vaccine is inversely associated? Currently the answer is not known but speculations can be made. One of the possible mechanisms may be the immune suppression that was experienced by most HD patients (10, 17). Specific antibody production after HBV vaccination is generated via B-cell activation by CD4+ Th1-helper (class II) and CD8+ CTL-cytotoxic T-cell (class I restricted T-cell) responses (18, 19). In this regard, it has been shown that monocyte function, cooperation and interaction between antigen presenting cells and CD4+ T cells are impaired in uremia. Moreover, dysregulation at the TCR/CD3 receptor level in uremia may result in an inadequate expression of adhesion and accessory or co stimulatory molecules, and thereby may cause the blunted signaling pathway (10, 20). A functional defect of the B7/D28 pathway could contribute to this effect, because in healthy people a single responsive haplotype inherited as a dominant trait is sufficient for a normal antibody response (21). In addition, dialysis patients have reduced cellular immunity, being attributable to reduced life span of lymphocytes, lymphocytopenia, lymphocyte transformation, and suppressor lymphocytes (17). In concordance with these findings, recently, Litjens et al. (22) and Armstrong et al. (23) have reported higher CD4+ counts to be associated with a higher likelihood of patients to develop an antibody response after hepatitis B vaccination. Very recently, it was shown that not only higher CD4+ lymphocyte count but CD4+/CD8+ ratio was also associated with higher seroconversion in HD patients who were vaccinated with HBV vaccine (17). As an interesting finding, the current study has shown that increased Hs-Crp levels was positively associated with seroconversion. Thus, in the light of all these findings. it could be speculated that because of depressed immunity, dialysis patients are not able to respond to hepatitis B vaccination, and when they respond, they have lower antibody titers and do not maintain adequate antibody levels over time (7).

The present study showed that blood urea nitrogen, creatinine and albumin levels were positively associated with the response to HBV vaccine. Previously, serum albumin levels were shown to be associated with better seroconversion in HD patients (1, 5, 24, 25). Thus, the current findings could be explained in the context of a relationship between better nutritional status and better vaccination response.

This study has limitations that deserve to be mentioned. Firstly, since the study has a retrospective design the reliability of potential confounders may be questioned. However, in our country it is mandatory to record data about demographics, laboratory parameters and vaccination status and these data are checked regularly by the ministry of health. Secondly, since the study has a cross-sectional design, the findings do not prove a cause and effect relationship. Additionally, reliable information about the brand of vaccines is not available and it is probable that vaccine brands are heterogeneous. Lastly, although no bleeding event was reported in the medical records of the patients during the study period, routine endoscopy or colonoscopy was not available.

In conclusion, EPO resistance was negatively associated with anti-HBs levels and seroconversion. More studies are needed to highlight the underlying mechanisms regarding EPO resistance and response to HBV vaccination in HD patients.
